# Effectiveness of a Web-Based Computer-Tailored Multiple-Lifestyle Intervention for People Interested in Reducing their Cardiovascular Risk: A Randomized Controlled Trial

**DOI:** 10.2196/jmir.5147

**Published:** 2016-04-11

**Authors:** Vera Storm, Julia Dörenkämper, Dominique Alexandra Reinwand, Julian Wienert, Hein De Vries, Sonia Lippke

**Affiliations:** ^1^ Department of Psychology and Methods Jacobs University Bremen Bremen Germany; ^2^ School for Public Health and Primary Care (CAPHRI) Department of Health Promotion Maastricht University Maastricht Netherlands; ^3^ Institute for Social Medicine and Epidemiology University of Lübeck Lübeck Germany; ^4^ Bremen International Graduate School for Social Sciences Bremen Germany

**Keywords:** Web-based intervention, computer tailoring, cardiovascular disease, habit strength, self-efficacy, planning

## Abstract

**Background:**

Web-based computer-tailored interventions for multiple health behaviors can improve the strength of behavior habits in people who want to reduce their cardiovascular risk. Nonetheless, few randomized controlled trials have tested this assumption to date.

**Objective:**

The study aim was to test an 8-week Web-based computer-tailored intervention designed to improve habit strength for physical activity and fruit and vegetable consumption among people who want to reduce their cardiovascular risk. In a randomized controlled design, self-reported changes in perceived habit strength, self-efficacy, and planning across different domains of physical activity as well as fruit and vegetable consumption were evaluated.

**Methods:**

This study was a randomized controlled trial involving an intervention group (n=403) and a waiting control group (n=387). Web-based data collection was performed in Germany and the Netherlands during 2013-2015. The intervention content was based on the Health Action Process Approach and involved personalized feedback on lifestyle behaviors, which indicated whether participants complied with behavioral guidelines for physical activity and fruit and vegetable consumption. There were three Web-based assessments: baseline (T0, N=790), a posttest 8 weeks after the baseline (T1, n=206), and a follow-up 3 months after the baseline (T2, n=121). Data analysis was conducted by analyzing variances and structural equation analysis.

**Results:**

Significant group by time interactions revealed superior treatment effects for the intervention group, with substantially higher increases in self-reported habit strength for physical activity (F1,199=7.71, *P*=.006, Cohen’s d=0.37) and fruit and vegetable consumption (F1,199=7.71, *P*=.006, Cohen’s d=0.30) at posttest T1 for the intervention group. Mediation analyses yielded behavior-specific sequential mediator effects for T1 planning and T1 self-efficacy between the intervention and habit strength at follow-up T2 (fruit and vegetable consumption: beta=0.12, 95% CI 0.09-0.16, *P*<.001; physical activity: beta=0.04, 95% CI 0.02-0.06, *P*<.001).

**Conclusions:**

Our findings indicate the general effectiveness and practicality of Web-based computer-tailored interventions in terms of increasing self-reported habit strength for physical activity and fruit and vegetable consumption. Self-efficacy and planning may play major roles in the mechanisms that facilitate the habit strength of these behaviors; therefore, they should be actively promoted in Web-based interventions. Although the results need to take into account the high dropout rates and medium effect sizes, a large number of people were reached and changes in habit strength were achieved after 3 months.

**Trial Registration:**

Clinicaltrials.gov NCT01909349; https://clinicaltrials.gov/ct2/show/NCT01909349 (Archived by WebCite at http://www.webcitation.org/6g5F0qoft) and Nederlands Trial Register NTR3706 http://www.trialregister.nl/ trialreg/admin/rctview.asp?TC=3706 (Archived by WebCite at http://www.webcitation.org/6g5F5HMLX)

## Introduction

Cardiovascular diseases (CVD) are major causes of morbidity and mortality in men older than 45 years and women older than 65 years in most developed European countries, including Germany and the Netherlands [1-5]. Regular physical activity and a healthy diet play an important role in preventing CVD because of their wide range of beneficial effects on physical health. Results from different meta-analyses reveal that people who already suffer from CVD have a lower risk of reinfarction [6], cardiac mortality [6-8] and overall mortality [6,7,9] if they improve their physical activity levels. Sufficient fruit and vegetable consumption is also recommended because it reduces the risk of further cardiovascular issues, such as coronary heart disease [10,11] and ischemic heart disease [12], cardiac mortality [13], and overall mortality [11].

To reduce CVD risk, one of the main goals is to adopt a healthier lifestyle (ie, regular physical activity and sufficient fruit and vegetable consumption). Changes that make these behaviors more habitual are a desired goal in primary and secondary prevention because once a behavior has become habitual it requires less conscious effort and relapses become less likely [14,15]. Habituation of the healthy behavior may be the final phase in the health behavior change chain, whereby the behavior has stabilized and its strength has plateaued [14].

People who are aware of their risk for CVD are usually highly motivated to practice recommended health behaviors and break old, unhealthy habits [16]. However, the process of health behavior change involves motivational factors that extend beyond merely having knowledge about behavior change benefits [17] and good intentions [18]. Even when people successfully initiate recommended changes, the gains are often short term and, without intervention, behavior change adherence declines over time [19,20]. Thus, long-term studies investigating the underlying mechanisms of health behavior maintenance are needed.

There is ample empirical support that intentions for behavioral change may best provoke behavior initiation by increasing the use of self-regulation strategies (ie, self-efficacy [21,22] and planning [23-25]). According to Bandura [26], self-efficacy describes optimistic self-beliefs concerning the ability to cope with possible failure and recover from relapses. Perceived self-efficacy seems to be important at all points in the health behavior change process [21] and is not only important for behavior initiation, but also behavior maintenance, recovery, and habituation. Evidence for the relevance of techniques that increase self-efficacy can be found in intervention studies, which found that experimentally induced changes in selfefficacy were positively associated with behavior initiation at a later point [27-29]. However, research on the direct effect on habit strength is limited.

In addition to self-efficacy, it is likely that planning promotes habit strength because habits are assumed to result from frequent behavior enactment in stable settings [30-32]. For example, if a person plans to go swimming on Fridays after dinner, the behavior becomes closely tied to contextual cues such as the time and location for which he or she chose to perform the initial action plan and the behavior becomes automatized with minimal forethought [15]. Previous intervention studies using self-regulatory techniques have revealed effects on habit strength at short-term follow-up in the case of physical exercise [33] and nonsmoking [34], although no research is available addressing multiple behaviors.

Because habit strength is a relatively new concept in behavioral intervention research, it is not yet fully understood how planning and self-efficacy might interplay with habit strength. Interventions that make use of both self-efficacy and planning techniques may enhance social cognitions, thereby leading to increased habit strength. Thus, mediation analysis might unfold the underlying working mechanisms of such an intervention.

A growing area of research focuses on the incorporation of the Internet as a mode of delivery to allow for individualized behavior change interventions [35-38]. Because interventions cannot fit all populations and circumstances in the same way, tailoring intervention content and offering personal behavioral and action feedback might increase the effectiveness of such programs in comparison to generic interventions or so-called “one-size-fits-all” approaches [39,40]. Specific tailored feedback for individuals based on their perceptions about a given behavior may be similar to feedback provided in face-to-face interactions and thus hold a higher personal relevance for the participant [41-43]. In addition, compared to face-to-face interventions, tailored interventions are easily accessible when delivered via the Internet and provide a cost-effective means to reach a wide population [35,36]. Previous studies on computer-tailored Web-based health behavior change interventions provided positive results for a variety of health behaviors, including physical activity [44-48], fruit and vegetable consumption [49,50], and multiple health behaviors [51-53] among the general adult population as well as people with cardiovascular risk profiles [54,55]. Although these previous Web-based computer-tailored studies focused on behavioral achievement, this study extended this topic by directly assessing its ability to enhance habit strength.

In this study, the first objective is to investigate the effects of an 8-week Web-based computer-tailored intervention on improvements of self-reported physical activity habit strength and fruit and vegetable consumption habit strength among people who were interested in reducing their cardiovascular risk. Moreover, we also test the prediction that social cognitive variables targeted by the tailored intervention (ie, self-efficacy and planning) increase more from the baseline in the intervention group than in the control group. Finally, we investigate whether changes in self-efficacy and action planning mediate the effect of the intervention on improvements in habit strength after two follow-up measurements. Testing the mechanisms of how the intervention exhibits an effect on proximal indicators of habit strength is the added value of our research. It is only when we know whether interventions work in terms of supporting habit formation by successfully targeting self-efficacy and planning by model learning and concrete planning tasks that we can conclude what online interventions should address in the future.

## Methods

A detailed description of the study protocol has been published previously [56]; therefore, only a summary of the study methodology and procedures is provided.

### Study Design, Procedure, and Participants

This study was a randomized controlled trial involving one intervention group and one waiting control group. There were three assessments: baseline (T0, N=790), a posttest at 8 weeks after the baseline (T1, n=206), and a follow-up 3 months after the baseline (T2, n=121). The waiting control group obtained access to the 8-week Web-based computer-tailored intervention at T2 after the intervention group had finished the intervention. The study received ethical approval by the Deutsche Gesellschaft für Psychologie in Germany (EK-A-SL022013) and the Medical Ethics Committee of Atrium Medical Centre Heerlen in the Netherlands (12-N-124).

Enrollment and follow-up took place from July 2014 to February 2015 in Germany and the Netherlands. We used different recruitment strategies: participants were recruited face-to-face by the authors of this study in 10 German and eight Dutch cardiac rehabilitation facilities and heart training groups. The authors of this study contacted the centers for acquisition and they were willing to participate. In addition, we called for participation via Internet platforms on diabetes and cardiovascular diseases as well as via an email invitation from two research agency online panels in Germany and the Netherlands. No data on how many participants were recruited through each strategy were available. The inclusion criteria were as follows: age between 20 and 85 years, no contraindications for physical activity and fruit and vegetable consumption, having an interest in improving physical activity and fruit and vegetable consumption, sufficient reading and writing skills in the relevant language (German or Dutch), and computer literacy and Internet access. Participation in the study was voluntary and data were anonymized.

**Figure 1:**
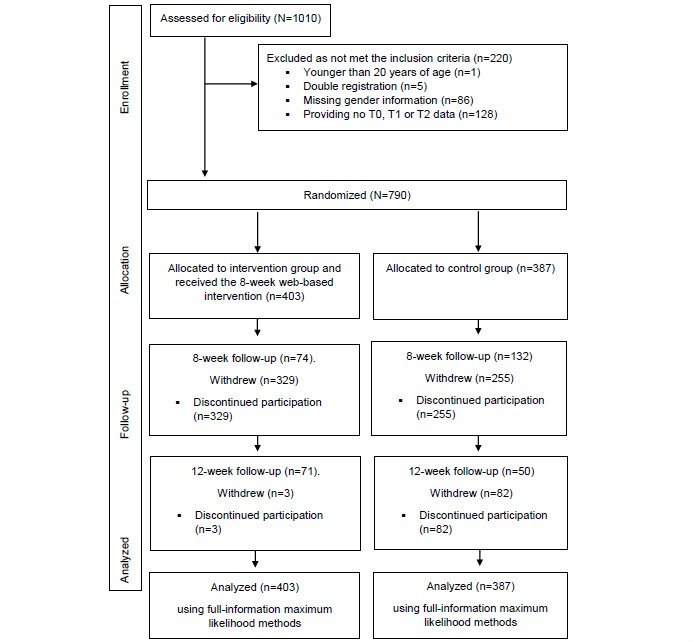
Flowchart of participants through the study.

Figure 1 shows the flow of participants from enrollment in the study to allocation to the two conditions (intervention group and waiting control group) and follow-up visits after 8 and 12 weeks. To obtain access to the Web-based questionnaires, the participants registered on the Rehabilitation-Aftercare for an optimal Transfer into Autonomous daily life (RENATA) website with a self-chosen nickname and password. The website was also open to the general public and provided broad information on the inclusion criteria and the procedure of the 8-week intervention as well as the duration of the questionnaires. Participants were made aware of the two-group design and the information provided was identical for all study participants, independent of the recruitment strategy.

After providing informed consent online, 1010 participants were then randomly assigned to either the intervention group or the waiting control group. Both groups took part in the identical baseline measurement (T0). Randomization into the intervention group and waiting control group was performed by the content management system, TailorBuilder, which was developed for Web-based tailored interventions. No block or cluster randomization was applied; rather, the randomization was conducted at the individual level. Participants and the authors of this study were blinded to their allocation for the duration of the study. The experiment was double blind. Overall, 220 datasets were excluded by the research team because of double registration (n=5), missing gender information (n=86), inadequate age (n=1 younger than 20 years), and no available T0, T1, or T2 data (n=128).

### Intervention Program

After registration, the 8-week intervention was delivered to the intervention group via the Internet, addressing physical activity in the first 4 weeks and fruit and vegetable consumption in the following 4 weeks. Once a week, the study participants were reminded to participate in the weekly intervention sessions and the follow-up measurements by automatically generated emails containing a link to the respective questionnaire.

The intervention was a Web-based computer-tailored intervention to increase physical activity and fruit and vegetable consumption among people who intended to change their physical activity and fruit and vegetable consumption. We used the Health Action Process Approach as a theoretical framework to develop the 8-week Web-based intervention [21,57]. The eight weekly sessions in the intervention period targeted the concepts of the different stages (nonintender, intender, and actor) via the use of behavior change techniques, such as providing information about behavioral risk, the benefits of behavior change, intention formation, barrier identification, prompting specific goal setting, and reviewing behavioral goals [58]. These techniques have proven effective in other computer tailoring programs [51,59]. Physical activity was discussed during the first four intervention sessions (sessions 1-4) and the last four sessions focused on fruit and vegetable consumption (sessions 5-8). In the following, we briefly describe the content of each session for both behaviors. In sessions 1 and 5, participants received tailored feedback about their risk perception, outcome expectancies, and their actual health behavior regarding physical activity and fruit and vegetable consumption based on their previous assessment. During the second and sixth sessions, participants were asked to determine personal goals and action plans for physical activity and fruit and vegetable consumption. During this session, participants received example plans and tailored feedback on how to structure a plan, and what elements a plan should contain (where, when, who, how long, with whom). Subsequently, participants had the opportunity to adapt their plans. Self-efficacy was also addressed during this time and the following sessions and participants were provided with motivating feedback on how to perform the desired behavior. During the third and seventh sessions, participants were asked whether they had succeeded in performing the action plan and if they would like to adjust the plan. People who indicated having problems formulating plans received role-model examples. Thereafter, coping planning was addressed in the third and seventh sessions, whereby participants were asked to identify personal barriers and generate coping plans. Again, these coping plans were evaluated during the next session and could be adjusted by the participants. The fourth and eighth sessions focused on social support. Participants developed an overview about people in their environment who could support them in achieving their plans. During the intervention, different types of feedback were provided. Ipsative feedback was used to provide participants with an overview of their development regarding physical activity and fruit and vegetable consumption during the intervention. This feedback was based on a short questionnaire that participants had to complete at the beginning of each session. Normative feedback was included to compare the participants’ behavior with the norm of the population. Bar charts were included in each session to present the progress of the participant’s behavior change. In this study, feedback and behavioral recommendations referred to the goals of five portions of fruit and vegetables a day and physical activity for at least 30 minutes five times a week because this constituted the best match for the target group [11,60]. Bar charts were included to monitor the behavior change progress and a personal tone was applied.

### Measurement Instruments

All variables were self-assessed online at baseline (T0) and after the 8-week intervention period (T1) and during the 3-month follow-up (T2). Study participants indicated all social cognitive items on Likert scales ranging from “1=not true” to “7=exactly true.”

### Sociodemographic Variables

We assessed sociodemographic information such as gender (1=male, 2=female), year of birth, country (1=Netherlands, 2=Germany), employment status (1=working part-time, 2=working full-time, 3=in training, 4=unemployed, 5=retired, 6=housewife/-husband), marital status (1=single, 2=close relationship but not living together, 3=close relationship and living together, 4=marital partnership/common law marriage, 5=divorced, 6=widowed), and highest level of education (1=no school graduation, 2=primary school education, 3=secondary school education, 4=vocational school graduation, 5=university entrance diploma, 6=other) in the baseline questionnaire. The participants additionally reported body height and body weight to calculate their body mass index (BMI) at T0, T1, and T2.

### Intentions

For physical activity, the three independent items used were “On 5 days a week for 30 minutes (or a minimum of 2.5 hours per week), I have the intention to perform...” (1) “strenuous physical activity,” (2) “moderate physical activity,” or (3) “walking activity” [59]. Intention about fruit and vegetable consumption was assessed using the item “I seriously intend to eat at least five portions of fruit and vegetable daily” [61].

### Self-efficacy

Physical activity self-efficacy [62] was assessed with five items (Cronbach alpha=.88), such as “I am certain that I can be physically active permanently at a minimum of 5 days a week for 30 minutes.” Self-efficacy for fruit and vegetable consumption [29] was assessed by five items (Cronbach alpha=.92), such as “I am certain that I can eat 5 portions of fruit and vegetable a day even if it is sometimes difficult.”

### Action Planning and Coping Planning

Action planning and coping planning were assessed using six items for physical activity (Cronbach alpha=.91) and six items for fruit and vegetable consumption (Cronbach alpha=.92). For both target behaviors, the question started with “For the next month, I have already planned in detail...” (1) “which physical activities I would like to do,” (2) “when I have to be especially cautious not to stop being active,” and (3) “what I can do in difficult situations to stick to my intentions” [63].

Planning for fruit and vegetable consumption started with the same phrase, followed by the three items: (1) “when I will eat 5 portions fruit and vegetables,” (2) “which fruit and vegetables I will eat,” and (3) “when I need to be especially cautious not to fall into my old eating habits” or “what I can do in difficult situations to stick to my intentions” [29].

### Habit Strength

The strength of habit for physical activity (Cronbach alpha=.88) and fruit and vegetable consumption (Cronbach alpha=.93) was measured with an abbreviated version of the Self-Report Habit Index (SRHI) [64] and included the two items “Being physically active for at least 30 minutes on 5 days a week is something that...” and “Eating five portions of fruit and vegetable per day is something...” (1) “has become a confirmed habit” and (2) “I do without thinking about it.”

### Statistical Analyses

Data analysis was conducted with SPSS version 22. Dropout analysis was performed using ANOVAs for the quantitative variables age, baseline intentions, baseline habit strength, and BMI. Chi-square tests were performed for the categorical variables gender and country. Age, gender, country, employment status, marital status, highest level of education, and BMI were included as covariates in all analyses because we were not interested in subgroup differences.

To investigate the effectiveness of the intervention, we first conducted separate ANCOVAs with repeated measures analyses for the four outcome measures for habit strength for regular physical activity, habit strength for fruit and vegetable consumption, self-efficacy, and planning. In each analysis, time, group, and a group by time interaction were entered as independent variables and the group by time interactions were interpreted. Effect sizes for differences in means are presented as Cohen’s *d*. Those effect sizes less than 0.30 were considered small, those between 0.30 and 0.80 were considered medium, and those larger than 0.80 were regarded as large [65].

To examine whether intervention effects on habit resulted from changes in social cognitive variables, we used mediation analysis to test indirect effects of intervention on change in habit strength through changes in those cognitions that the intervention aimed to modify. The mediation analyses were performed using SPSS AMOS mediation models according to Preacher and Hayes [66]. The bootstrapping approach (5000 bootstrap samples) was used to estimate 95% confidence intervals of the standardized effects of the intervention on habit strength through self-efficacy and planning.

Baseline habit strength and baseline levels of self-efficacy and planning were controlled for. The level of statistical significance was set at *P*<.05. All reported *P* values are two-tailed. We used no statistical measures to correct for multiple testing.

For the 16 variables used in the analyses, the missing data proportions were <21% at T0, <19% at T1, and <17% at T2. Therefore, missing study variables were estimated with the full-information maximum likelihood (FIML) method. We exported the estimated data to SPSS to perform further analyses. FIML is based on the maximum likelihood algorithm and, compared with other options (ie list-/pairwise deletion, regression imputation), maximum likelihood estimates exhibit the least bias [67]. For example, Demirtas et al [68] reported that parameters were estimated accurately with missing rates up to 25%.

## Results

### Participation and Sample Characteristics

The final sample consisted of 790 persons with a mean age of 50.9 years at baseline (SD 12.2, range 20-84). In all, 62.9% (497/790) of the participants were female, 71.8% (646/790) of the participants were married or in a relationship, and 569 participants (72.0%) were employed either full- or part-time. The mean BMI was 27.6 (SD 5.5, range 15.0-60.8), indicating that the participants as a group were considered overweight. Table 1 provides an overview of the main baseline variables in this study.

The *t* tests revealed small yet significant differences between German (n=371) and Dutch participants (n=419) regarding age (*t*_789_=8.51, *P*=.004), BMI (*t*_789_=5.38, *P*=.02), self-efficacy for physical activity (*t*_789_=8.24, *P*=.004), and planning for fruit and vegetable consumption (*t*_789_=18.91, *P*<.001). German participants were found to be slightly older and have a higher BMI and higher self-efficacy levels compared to the Dutch participants, whereas they reported less planning of their fruit and vegetable consumption. In addition, there were more women among the Dutch participants (χ^2^_790_=9.1, *P*=.004).

### Dropout Analyses

Dropout analyses (1=dropout, 2=no dropout) showed no significant differences between the participants who completed all waves of data collection (T0, T1, and T2) and those who dropped out after T0 regarding age (*F*_1,789_=1.11, *P*=.29), BMI (*F*_1,789_=0.38, *P*=.54), baseline intentions (fruit and vegetable consumption: *F*_1,789_=0.81, *P*=.78; physical activity: *F*_1,798_=0.02, *P*=.90), and baseline habit strength (fruit and vegetable consumption: *F*_1,789_=0.23, *P*=.63; physical activity: *F*_1,789_=2.75, *P*=.10). In addition, the dropouts after T0 did not significantly differ from those who participated in the follow-up questionnaire in terms of gender (χ^2^_790_=2.1, *P*=.15) and country (χ^2^_790_=1.2, *P*=.27): Men and women, as well as German and Dutch participants dropped out after the baseline measurement T0 in equal numbers.

Those who dropped out after posttest T1 did not differ from those who participated in all measurement points in terms of age (*F*_1,789_=3.36, *P*=.07), BMI (*F*_1,789_=0.42, *P*=.52), baseline intentions (fruit and vegetable consumption: *F*_1,789_=1.56, *P*=.21; physical activity: *F*_1,798_=0.01, *P*=.98), and baseline habit strength for fruit and vegetable consumption (*F*_1,789_=1.22, *P*=.27). In addition, the dropouts after T1 did not differ from those who participated in the follow-up questionnaire in terms of country (χ^2^_790_ =1.2, *P*=.27); German and Dutch participants dropped out after the baseline measurement T0 in equal numbers. However, there were significant differences between those who dropped out after T1 and those who completed all measurement points in terms of baseline habit strength for physical activity (*F*_1,789_=6.71, *P*=.01) and gender (χ^2^_790_=4.3, *P*=.04). Slightly more women than men dropped out after T1; those people who dropped out after T1 showed significantly lower baseline habit strength for physical activity than those who completed all measurement points. Overall, T0 to T1 attrition was 73.9% (584/790) and T0 to T2 attrition was 85.3% (705/790).

Table 2 presents the number of participants who participated in the single intervention sessions. Participation declined from 90.8% (314/790) in the first session to 19.9% (69/790) participation in the last session of the 8-week intervention. Participants completed a mean 2.0 (SD 2.4) intervention sessions of eight potential sessions. Most participants completed only one session (41.9%, 331/790), 15.3% (120/790) completed two sessions, 8.7% (68/790) completed three sessions, 4.6% (36/790) completed four sessions, 3.8% (30/790) completed five sessions, 5.8% (45/790) completed six sessions, 5.5% (43/790) completed seven sessions, and 8.1% (63/790) completed all eight sessions.

### Intervention Effects on Baseline to Posttest Changes in Habit Strength

The assumption that the 8-week Web-based intervention would lead to an increase in habit strength for fruit and vegetable consumption and physical activity at posttest T1 was tested first. The results of the ANCOVA with repeated measurements (see Table 3) showed an interaction effect of group×time for habit strength for fruit and vegetable consumption (*F*_1,199_=7.71, *P*=.006, Cohen’s *d*=0.30) as well as habit strength for physical activity (*F*_1,199_=7.71, *P*=.006, Cohen’s *d*=0.37) with medium effect sizes. The intervention group showed a higher increase of dietary habit strength and physical activity habit strength from baseline (T0) to posttest (T1) than the waiting control group. This was then tested for the T2 follow-up, examining changes from T0 to T2. There was neither an intervention effect for habit strength for fruit and vegetable consumption (*F*_1,114_=0.82, *P*=.14) nor for habit strength for physical activity (*F*_1,114_=0.43, *P*=.24) at follow-up T2.

**Table 1:** Sample characteristics at baseline T0 (N=790).

Characteristics		Total (N=790)	Waiting control group (n=387)	Intervention group (n=403)
Age (years), mean (SD)		50.8 (12.2)	50.8 (12.3)	50.9 (12.0)
Gender, n (%)				
	Male	293 (73.1)	151 (51.5)	142 (48.5)
	Female	497 (62.9)	236 (47.5)	261 (52.5)
BMI (kg/m^2^), mean (SD)		27.6 (5.4)	27.3 (5.2)	27.8 (5.6)
Physical activity, mean (SD)				
	Intentions	3.9 (1.0)	3.9 (1.0)	4.0 (0.9)
	Planning	4.3 (1.4)	4.3 (1.4)	4.3 (1.5)
	Self-efficacy	4.6 (1.3)	4.5 (1.4)	4.7 (1.3)
	Habit strength	3.5 (1.8)	3.4 (1.7)	3.6 (1.9)
Fruit and vegetable consumption, mean (SD)				
	Intentions	4.5 (1.4)	4.5 (1.4)	4.6 (1.3)
	Planning	3.8 (1.5)	3.7 (1.4)	3.8 (1.6)
	Self-efficacy	4.7 (1.5)	4.6 (1.3)	4.7 (1.5)
	Habit strength	3.7 (1.9)	3.6 (1.8)	3.8 (1.9)
Ethnicity, n (%)				
	German	371 (47.0)	189 (50.9)	182 (49.1)
	Dutch	419 (53.0)	198 (47.3)	221 (52.7)
Educational level, n (%)				
	No school graduation	1 (0.1)	0 (0.0)	1 (100.0)
	Primary school education	23 (2.9)	15 (65.2)	8 (34.8)
	Secondary school education	86 (10.9)	40 (46.5)	46 (53.5)
	Vocational school graduation	378 (47.8)	192 (50.8)	186 (49.2)
	University entrance diploma	242 (30.6)	112 (46.3)	130 (53.7)
	Other	60 (7.6)	28 (46.7)	32 (53.3)
Working situation, n (%)				
	Working full-time	396 (50.1)	191 (48.2)	205 (51.8)
	Working part-time	173 (21.9)	91 (52.6)	82 (47.4)
	Schooling/vocational training	15 (1.9)	8 (53.3)	7 (46.7)
	Unemployed	49 (6.2)	22 (44.9)	27 (55.1)
	Retired	115 (14.6)	59 (51.3)	56 (48.7)
	Housewife/-husband	42 (5.3)	16 (38.1)	26 (61.9)
Family status, n (%)				
	Single	78 (9.9)	35 (44.9)	43 (55.1)
	Close relationship but not living together	46 (5.8)	22 (47.8)	24 (52.2)
	Close relationship and living together	76 (9.6)	41 (53.9)	35 (46.1)
	Marital partnership/common law marriage	524 (66.3)	258 (49.2)	266 (50.8)
	Divorced	54 (6.8)	27 (50.0)	27 (50.0)
	Widowed	12 (1.5)	4 (33.3)	8 (66.7)

**Table 2:** Intervention use in terms of participation in the single sessions for physical activity and fruit and vegetable consumption.

Weekly intervention session		Participation in the specific session, n (%)
Physical activity		
	Session 1	373 (47.2)
	Session 2	240 (30.4)
	Session 3	202 (25.6)
	Session 4	148 (18.7)
Fruit and vegetable consumption		
	Session 5	166 (21.0)
	Session 6	141 (17.8)
	Session 7	132 (16.7)
	Session 8	123 (15.6)

**Table 3:** Changes of outcome measures from baseline (T0) to posttest (T1) (N=790).

Measure		Intervention group, mean (SD)	Waiting control group, mean (SD)	*F*_1,199_	*P*	Cohen’s *d*
Physical activity						
	Self-efficacy	0.22 (1.47)	–0.06 (1.28)	2.11	.01	0.22
	Planning	0.60 (1.76)	0.14 (1.25)	5.70	.02	0.35
	Habit strength	1.00 (1.66)	0.34 (1.53)	7.71	.006	0.37
Fruit and vegetable consumption						
	Self-efficacy	0.22 (1.47)	–0.06 (1.28)	1.40	.03	0.20
	Planning	0.58 (1.72)	0.03 (1.60)	5.48	.02	0.36
	Habit strength	0.83 (1.83)	0.26 (1.51)	7.71	.006	0.30

### Intervention Effects on Baseline-Posttest Changes in Self-Efficacy and Planning

An ANCOVA with repeated measures revealed significant interaction effects of condition×time for self-efficacy for physical activity (*F*_1,199_=2.11, *P*=.01, Cohen’s *d*=0.22) and self-efficacy for fruit and vegetable consumption (*F*_1,199_=1.40, *P*=.04, Cohen’s *d*=0.20). The increase in self-efficacy from baseline (T0) to posttest (T1) was higher in the intervention group in comparison to the waiting control group. There was no intervention effect for self-efficacy for fruit and vegetable consumption (*F*_1,114_=3.63, *P*=.06) nor for self-efficacy for physical activity (*F*_1,114_=0.39, *P*=.54) at follow-up T2. For planning, we found a significant interaction effect of condition×time for both physical activity (*F*_1,199_=5.70, *P*=.02, Cohen’s *d*=.35) and fruit and vegetable consumption (*F*_1,199_=5.48, *P*=.02, Cohen’s *d*=0.36) with small to medium effect sizes. This indicates that the intervention led to a significantly higher increase in planning from baseline (T0) to posttest (T1) in the intervention group as compared to the waiting control group for both target behaviors.

### Mediation Analyses

**Figure 2:**
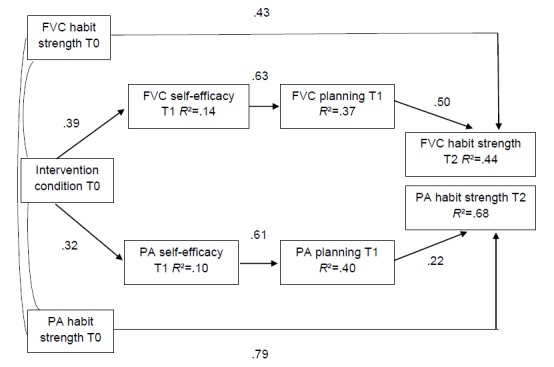
Conceptual model with standardized regression coefficients showing the effect of the intervention for fruit and vegetable consumption (FVC) and physical activity (PA) habit strength at follow-up controlling for age, gender, employment status, highest education, marital status, country, BMI, and baseline levels for self-efficacy and planning.

To address whether the intervention had an effect on habit strength through self-efficacy and planning, self-efficacy and planning at posttest T1 were considered to serve as sequential mediators between the intervention and habit strength at T2 follow-up. The entire hypothesized model is portrayed in Figure 2 and shows an acceptable fit to the data (χ^2^_190_=6.1, *P*<.001; comparative fit index=.91; Tucker-Lewis index=.81; root mean square error of approximation=.08). The intervention group condition significantly predicted T1 self-efficacy (physical activity: beta=0.32, *P*<.001; fruit and vegetable consumption: beta=0.39, *P*<.001), holding higher mean values in the intervention group. T1 self-efficacy was significantly interrelated with T1 planning (physical activity: beta=0.61, *P*<.001; fruit and vegetable consumption: beta=0.63, *P*<.001) for both target behaviors, whereas T1 planning predicted subsequent T2 habit strength (physical activity: beta=0.22, *P*<.001; fruit and vegetable consumption: beta=0.50, *P*<.001). Accordingly, people who planned more were also more likely to show strengthened habits later. Baseline habit strength also significantly predicted habit strength at T2 (physical activity: beta=0.79, *P*<.001; fruit and vegetable consumption: beta=0.43, *P*<.001). The standardized indirect effect of the intervention through T1 self-efficacy and T1 planning on T2 habit strength was beta=0.04 (95% CI 0.02-0.06) for physical activity and beta=0.12 (95% CI 0.09-0.16) for fruit and vegetable consumption. The multiple mediator model accounted for 68% of the variance in T2 physical activity habit strength and 44% of the variance in T2 fruit and vegetable consumption habit strength.

## Discussion

### Principal Results

The aim of this study was to test the effectiveness of a Web-based intervention in terms of improving habit strength for regular physical activity and fruit and vegetable consumption. The intervention led to significant increases in participants’ self-reported physical activity habit strength as well as fruit and vegetable consumption habit strength 8 weeks after baseline. This is in line with the results from previous self-regulatory intervention studies, which yielded effects on habit strength with a short-term followup in the case of physical exercise [33] and smoking cessation [34]. However, our study extends these findings to the context of Web-based computer-tailored interventions for physical activity and also fruit and vegetable consumption, and shows the online practicality of a multiple behavior change intervention.

Previous research has mainly tested how habit strength is formed based on cues to action [14,15]. Such a cue can be medical treatment or advice received during an eHealth program. Instead, we investigated which self-regulatory mechanisms accounted for the effect of the intervention on habit strength. We were able to show that the intervention successfully addressed two key intervention variables, self-efficacy and planning, which subsequently mediated the intervention effect on habit strength 3 months after the baseline (ie, changes in self-efficacy and planning contributed additively to habit strength). This replicates the results of Fleig et al [31], who used a multiple health behavior model and theoretical assumptions [69]. Both self-efficacy and planning were predictive of habit strength at a later point; thus, they are not only important for behavior initiation [27-29], but also behavior maintenance. In future studies, this behavior maintenance should be researched in more depth with an Internet study design in a medical context (ie, with patients only).

The theoretical framework of habit strengthening used in this study may also be applied to processes when individuals try to break unhealthy habits, such as smoking and snacking. For example, Webb et al [34] showed that smokers with moderate or low smoking habits were successful in overriding their unhealthy habitual responses when accompanied with behavioral alternatives that they had specified in an action plan (eg, “If I am walking from the office to my car, then I will chew some gum instead of smoking a cigarette!”). These compensatory cognitions were not researched explicitly in this study, but should be addressed in the future [69].

Our results for the hypothesized mechanisms are important because they point toward the potential target constructs of Web-based interventions and how to make such interventions more efficient. It is imperative to address self-efficacy and planning to enable individuals to develop habits and translate intentions into behaviors. However, in future research this needs to be evaluated further. For instance, it should be tested whether the intervention effect only translates in the sequence via self-efficacy first and then planning or whether it could also be that self-efficacy moderates the mediation of intentions into behavior via planning. In addition, experimental designs should test whether the intervention addressing planning works only in intenders or people with high self-efficacy because it was found in previous studies [63].

### Limitations

This study is subject to some limitations, including the measurement of our criterion variables fruit and vegetable consumption and physical activity habit strength. The SRHI offers a standardized and reliable measure to assess habituated action with evidence across behaviors and populations [70,71]. However, we relied on a short version of the habit strength measure referring to general physical activity and fruit and vegetable consumption. Future studies may include additional items of the SRHI to capture further facets of habitual automaticity (eg, lack of control). In addition, self-report for behavioral outcome measures of intervention studies can be criticized for their limitations, such as response and recall bias, underreporting, socially desirable answers, and measurement errors [72]. Thus, the inclusion of measures such as biomarkers or pedometers is advocated as an objective indicator of effectiveness [73,74].

Furthermore, the high dropout in our study needs to be addressed. Web-based interventions typically come with dropout rates [75] that can be very high (eg, up to 86% [76]). Although appropriate usage of the intervention differs among certain participant characteristics [76], we did not find any personal characteristics that could explain high dropout. Due to our widespread recruitment strategies, it can be assumed that a large number of the participants who signed up for the intervention did so out of curiosity rather than having a genuine interest in changing their health behavior, which could be one explanation for the high dropout rate [77]. Furthermore, dropout and not responding to questions could also be caused by intervention characteristics, such as the length of the questionnaire, layout, or navigation difficulties through the intervention [78]. Future studies should further investigate characteristics of dropout and nonresponse to eHealth interventions and consider how to tackle them to obtain larger sample sizes, including at follow-up. The results from a recent systematic review [79] show that the differences in technology and interaction predict user adherence in Web-based interventions.

One possible recommendation for future interventions is the inclusion of social media interaction, the integration of environmental components, and regular updates to promote adherence. Participants might show higher levels of engagement and complete program challenges in a Web-based program when they have social ties [80], the possibility to exchange experiences with others, and receive social support [81]. In addition, because environmental intervention components (eg, information on how to plan a cycling route for being physically active) might support people in finding possibilities to translate their goal intentions to behaviors [44], the integration of these environmental components could be useful in stimulating more active intervention participation.

Finally, our study participants form a rather heterogeneous group because we included participants via different recruitment channels. Unfortunately, no data on how many participants were recruited through each strategy are available. Although baseline intentions and sociodemographic data were controlled for in all analyses, a physician rating, medical diagnosis, or objective index of medical severity should be included as a control in future studies. In addition, there were small yet significant differences between German and Dutch participants regarding the measures used, although it is assumed that none of the differences in the results are due to country given that both countries have similar nutrition recommendations [82,83] and prevention campaigns (eg, “5 a day”) [84,85].

### Conclusion

The results of this study are important for the future development of Web-based computer-tailored interventions to improve lifestyle behaviors that can reduce the risk of cardiovascular events. Web-based computer-tailored interventions can be a suitable delivery mode to successfully foster changes in self-efficacy and planning, which predict physical activity and fruit and vegetable consumption habit strength. This research adds to the growing literature on real-world habit strengthening, with our findings suggesting that planning and confidence in one’s action may aid the process of making behavior automatic. Our results add up to the current body of knowledge because they display mechanisms of how this intervention affects behavioral habit change. Future interventions should address habit formation by targeting self-efficacy and planning by model learning and concrete planning tasks.

Due to the high dropout rate, our results must be interpreted with caution, although these findings can guide further research. In particular, the investigated constructs and mechanisms should be further elaborated. Practical implications can be retrieved from the fact that habit-strengthening resources for plans and self-efficacy boosters can be delivered briefly via the Internet, are easy for people to implement, and theoretically have the potential for longer-term impact.

Future interventions may benefit from aids to support the creation and recall of plans, particularly when accompanied with self-efficacy prompting techniques, such as vicarious experience, personalized feedback, providing contingent rewards, self-monitoring (tracking one’s own food- and exercise-related behavior), and becoming conscious of mastery experience [86,87].

## Abbreviations

BMI: body mass index
CVD: cardiovascular disease
FIML: full-information maximum likelihood
RENATA: Rehabilitation-Aftercare for an optimal Transfer into Autonomous daily life
SRHI: Self-Report Habit Index

## Appendix

Multimedia Appendix 1 CONSORT eHealth Checklist.
